# High Phenotypic Plasticity in a Prominent Plant Invader along Altitudinal and Temperature Gradients

**DOI:** 10.3390/plants10102144

**Published:** 2021-10-09

**Authors:** Rodolfo Gentili, Roberto Ambrosini, Benno A. Augustinus, Sarah Caronni, Elisa Cardarelli, Chiara Montagnani, Heinz Müller-Schärer, Urs Schaffner, Sandra Citterio

**Affiliations:** 1 Department of Earth and Environmental Sciences, University of Milano-Bicocca, Piazza della Scienza 1, 20126 Milano, Italy; sarah.caronni@unimib.it (S.C.); elisa.cardarelli@unimib.it (E.C.); chiara.montagnani@unimib.it (C.M.); sandra.citterio@unimib.it (S.C.); 2 Department of Environmental Science and Policy, University of Milano, Via Celoria 2, 20133 Milano, Italy; roberto.ambrosini@unimi.it; 3 Swiss Federal Institute for Forest, Snow and Landscape Research WSL, Zuercherstrasse 111, CH-8903 Birmensdorf, Switzerland; benno.augustinus@wsl.ch; 4 Department of Biology, University of Fribourg, Chemin du Musée 10, CH-1700 Fribourg, Switzerland; heinz.mueller@unifr.ch; 5 CABI, Rue des Grillons 1, CH-2800 Delémont, Switzerland; u.schaffner@cabi.org

**Keywords:** growth curve, plant traits, elevation gradient, climate change, invasive plant species, *Ophraella communa*, invasive species management

## Abstract

Studies on plant growth and trait variation along environmental gradients can provide important information for identifying drivers of plant invasions and for deriving management strategies. We used seeds of the annual plant invader *Ambrosia artemisiifolia* L. (common ragweed) collected from an agricultural site in Northern Italy (226 m. a.s.l; Mean Annual Air Temperature: 12.9 °C; precipitations: 930 mm) to determine variation in growth trajectories and plant traits when grown along a 1000-m altitudinal gradient in Northern Italy, and under different temperature conditions in the growth chamber (from 14/18 °C to 26/30 °C, night/day), using a non-liner modeling approach. Under field conditions, traits related to plant height (maximum height, stem height, number of internodes) followed a three-parameter logistic curve. In contrast, leaf traits (lateral spread, number of leaves, leaf length and width) followed non-monotonic double-Richards curves that captured the decline patterns evident in the data. Plants grew faster, reaching a higher maximum plant height, and produced more biomass when grown at intermediate elevations. Under laboratory conditions, plants exhibited the same general growth trajectory of field conditions. However, leaf width did not show the recession after the maximum value shown by plants grown in the field, although the growth trajectories of some individuals, particularly those grown at 18 °C, showed a decline at late times. In addition, the plants grown at lower temperatures exhibited the highest value of biomass and preserved reproductive performances (e.g., amount of male inflorescence, pollen weight). From our findings, common ragweed exhibits a high phenotypic plasticity of vegetative and reproductive traits in response to different altitudes and temperature conditions. Under climate warming, this plasticity may facilitate the shift of the species towards higher elevation, but also the in situ resistance and (pre)adaptation of populations currently abundant at low elevations in the invasive European range. Such results may be also relevant for projecting the species management such as the impact by possible biocontrol agents.

## 1. Introduction

Natural climatic variations associated with altitude are widely used to infer possible plant trait adaptations to temporal climate change and their phenotypic plasticity [1]. In response to increasing altitude and decreasing temperature, plants may modulate their functional traits including morphology, reproduction, and physiology [2,3,4,5]. With increasing altitude, the need of plants to survive and maintain reproductive success may result in either local adaptation or plastic responses [6,7]. To face sudden environmental changes, generally plastic response is very important over the short period whereas genetic responses is observed over longer-term periods [8]. Species showing larger plasticity may be better prepared to persist in new or stressing environments, helping the expansion of their geographical or altitudinal range across different environmental conditions [9,10], finally favoring local adaptation. Usually, along an altitudinal gradient, plants on higher sites invest a larger amount of resources in vegetative growth, with a possible reduction of reproductive output [7,11]. Plants often cope with resource deficit due to short vegetative seasons allocating biomass to resource-capturing organs (i.e., leaves and roots) [12]. Such ability has been demonstrated in plants in response to different levels or deficits of light, nutrient, water, and CO_2_ [7]. Along an altitudinal gradient, the study of these plant traits, which are highly sensitive to climate changes, can be also used to phenotypically ‘track’ the observed climatic variations [1,12].

In biological invasion studies, a high phenotypic plasticity of alien plants is widely acknowledged to contribute to invasion success even in harsh environments, often outcompeting native species [13,14]. Trait plasticity can explain the reason why some invasive species show better ability to establish in a wide range of environments, thanks to their aptitude to increase and/or maintain fitness in both favorable and stressful situations [15,16]. For instance, fast growth rate and modulation of reproductive periods allow alien plant species to establish over wide altitudinal and temperature ranges [14]. Recent studies in this direction have shown that an increasing number of alien species occur at higher altitudes in temperate regions, favored by warmer temperatures [17]. Particularly, acclimation to stressful conditions in adverse climatic circumstances is considered a key factor of the success of alien species colonization [18]. While a higher tolerance of warmer temperatures is supposed to be the key for a successful alien invader compared to native species, a growing number of studies also report better abilities of aliens to cope with low temperatures [19,20]. Alien species tend to acclimate to new areas also under harsh environmental conditions by modulating their growth traits such as plant height, number of vegetative shoots, and number of flowers [21]. For such reasons, some invasive species may be more adapted to climate change due to traits that facilitate rapid range shifts (e.g., resource allocations, short time to maturity) and their wide climatic tolerances [22].

Incorporating impacts of climate change into invasive species management has been identified as a priority for land managers [23]. Such a goal can be achieved by implementing prevention actions and strategic planning, adjusting control actions, and by information exchanges between researchers and managers [23,24]. Some specific plant traits modulated by temperature changes or new environmental conditions (for species colonizing new territories or higher altitudes) via phenotypic plasticity can be responsible for the invasion success of alien species. In a changing climate, trait plasticity could confer a strong competitive advantage to alien invaders compared to native species, therefore augmenting their impact on ecosystems [25]. For instance, plasticity to new environmental conditions is expected to influence management strategies using biocontrol agents that are generally dependent on plant phenology [26]. Indeed, life cycle and timing of releases of host-specific insects may greatly vary in relation to climatic condition, becoming asynchronous with respect to reproductive events, possibly reducing the success of biocontrol [27].

Despite investigations on how invasive alien plants adapt to altitudinal gradients can be important to the understanding of processes involved in their establishment and spreading, relatively little is known about their growth patterns, especially for herbaceous species. Most studies have focused on factors affecting primary production (e.g., respiration, photosynthesis, carbon fluxes), with scarce attention dedicated to resource allocation and turnover [28]. Recently, Kühn et al. [29] investigated the variability of plant functional traits along elevation gradients. They found that within-population variability of leaf traits decreased with altitude. March-Salas and Pertierra [14] monitored the phenological development of two invasive alien species (*Poa annua* and *Cerastium fontanum*) at different altitudes in a sub-Antarctic region; the species showed great acclimation (growth) and reproductive ability also under limiting conditions. Alexander et al. [30] investigated growth trends and reproductive traits in native and invaded ranges of eight invasive Asteraceae forbs along an altitudinal gradient; plants exhibited smaller size and fewer inflorescences towards higher altitudes.

Among invasive alien species, common ragweed (*Ambrosia artemisiifolia* L.) is a successful invader of great concern in Europe and around the world [31]. Since the 19th century, this species native to North America has been inadvertently introduced in Europe (and then in other continents) where it has established and has become a serious threat to both agriculture, economy, and human health due to the production of large amounts of highly allergenic pollen [31,32]. In Europe, some 13.5 million people suffer from ragweed-induced allergies, with an annual economic cost of approximately 7.4 billion Euros [33]. It is a fast-growing annual weed that, thanks to its wide ecological amplitude and high within-population genetic diversity, colonizes a large variety of open-disturbed habitats including crop and abandoned fields, roadsides, and ruderal areas [34,35,36]. In addition to flat temperate areas, the species has been also reported to colonize a wide range of climates along latitudinal and altitudinal gradients, from the sea level to mountains [37,38].

In this study, we aimed to investigate how variation in altitude and temperature affects phenotypic expression of growth-related and reproductive traits of this invasive alien plant. We grew common ragweed plants both in the field along an altitudinal gradient and in the laboratory under controlled conditions and used prediction models to estimate the species performance in relation to altitude and temperature. In particular, by fitting parametric growth curves with nonlinear mixed models (NLMMs), we tested for trait size and reproductive performance reduction of the individuals along to a ~1000 m altitudinal (at several sites) and a decreasing temperature gradient.

## 2. Results

### 2.1. Growth Trajectories and Biomass of Plant Grown in Field Conditions

**Figure 1:**
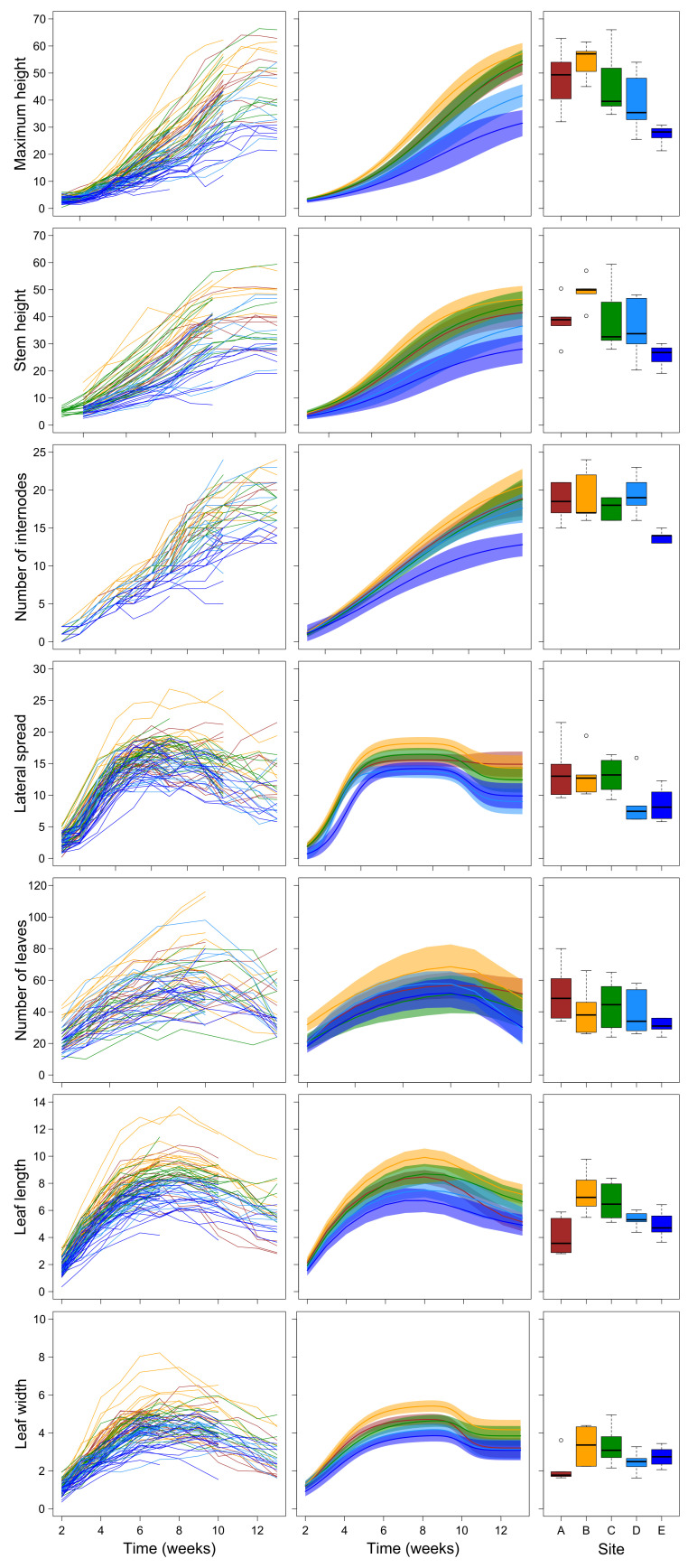
Left panels show growth trajectories of individual plants grown in the field (after the removal of deviant data points). Each line represents an individual plant. Central panel shows the interpolated growth curves and their 95% confidence intervals. Right panels show boxplots of the values of each plant measured at the last date (week 13). In all panels, colors represent sites (brown = site A, yellow = site B, green = site C, turquoise = site D, blue = site E).

Under field conditions, maximum height, stem height, and the number of internodes grew monotonically along the whole time span considered and their growth trajectories were best described by three-parameter logistic curves. In contrast, lateral spread, number of leaves, and leaf length and width followed non-monotonic double-Richards curves that captured the recession patterns evident in the data (Table 1; Figure 1). With the only exception of the number of leaves, all measured features showed significant differences among sites in the upper asymptote (K parameter).

For the number of leaves, differences appeared in the decrease after the maximum number, which was less marked in the lowest-altitude site A than in the other sites. Generally, plants at the highest altitude sites D and E showed lower values than those at the lower sites (Table 1; Figure 1). Leaf length and width were exceptions in this pattern, as plants at the lowest altitude site A showed lower values than those at sites D and E, while sites at intermediate altitude, particularly site B, showed higher values. (Table 1; Figure 1). Growth trajectories of stem height also showed differences in the scale parameter (reciprocal of the growth rates), generally denoting a trend toward slower growth rates at sites at increasing altitudes (Table 1; Figure 1). Notably, the lateral spread showed a very complex pattern of variation among sites, with all the parameters of the double-Richards curve differing significantly among sites (Table 1). However, plants at the highest-altitude site E showed a generally slower growth and reached lower maximum sizes than those at sites A, B, and C. Plants at site D initially grew similarly to plants at sites A, B, and C, but then they reached lower maximum and final values, similar to those of plants in site E (Figure 1).

Dry and wet biomass of plants showed significant differences among sites at different times. The growth patterns also differed among sites, as indicated by the significant site by time interaction (Table 2).

**Figure 2:**
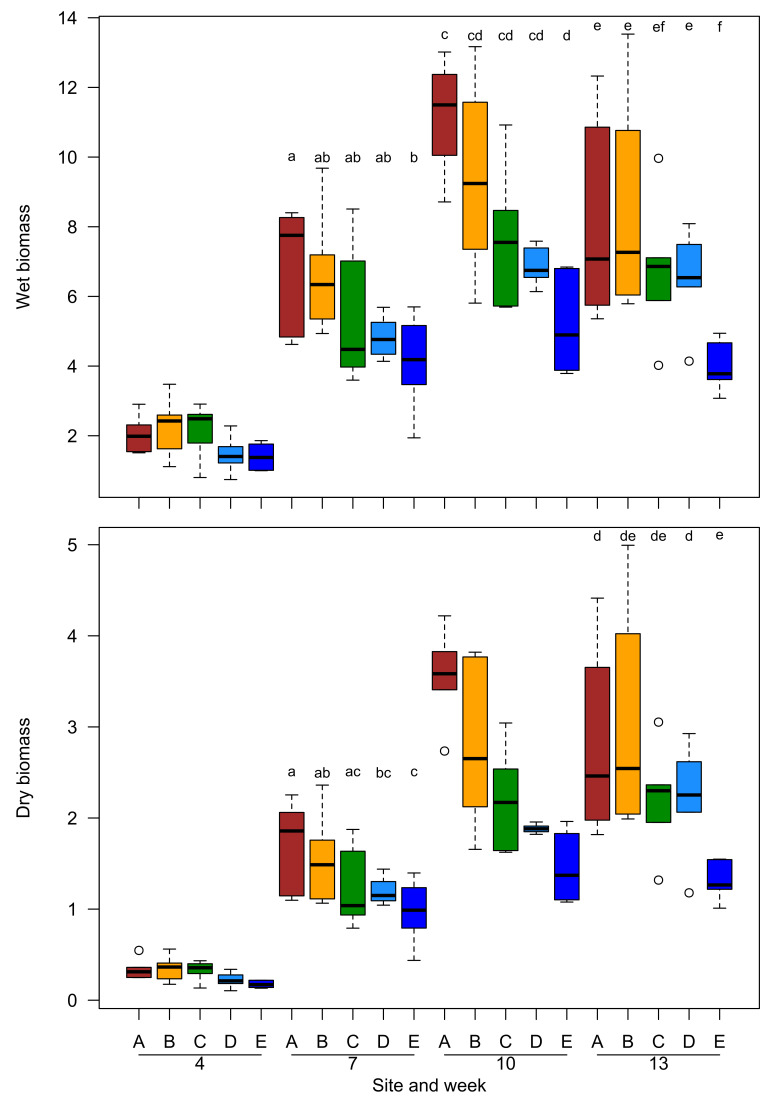
Boxplot of wet (upper panel) and dry (lower panel) biomass of plants grown at different sites (4, 7, 10, and 13 weeks). Different letters above bars denote significant differences between sites at that time. Circles represent outliers.

Both dry and wet biomass did not differ among sites at week 4, but plants at site E showed lower values than site A already at week 7, while the other sites showed intermediate values (Figure 2). The same general pattern remained until week 13. For dry biomass, difference among sites were not significant at week 10 due to large spread of values, although the median values showed the same pattern as above (Figure 2).

### 2.2. Growth Trajectories of Plants Grown in Laboratory Conditions

**Figure 3:**
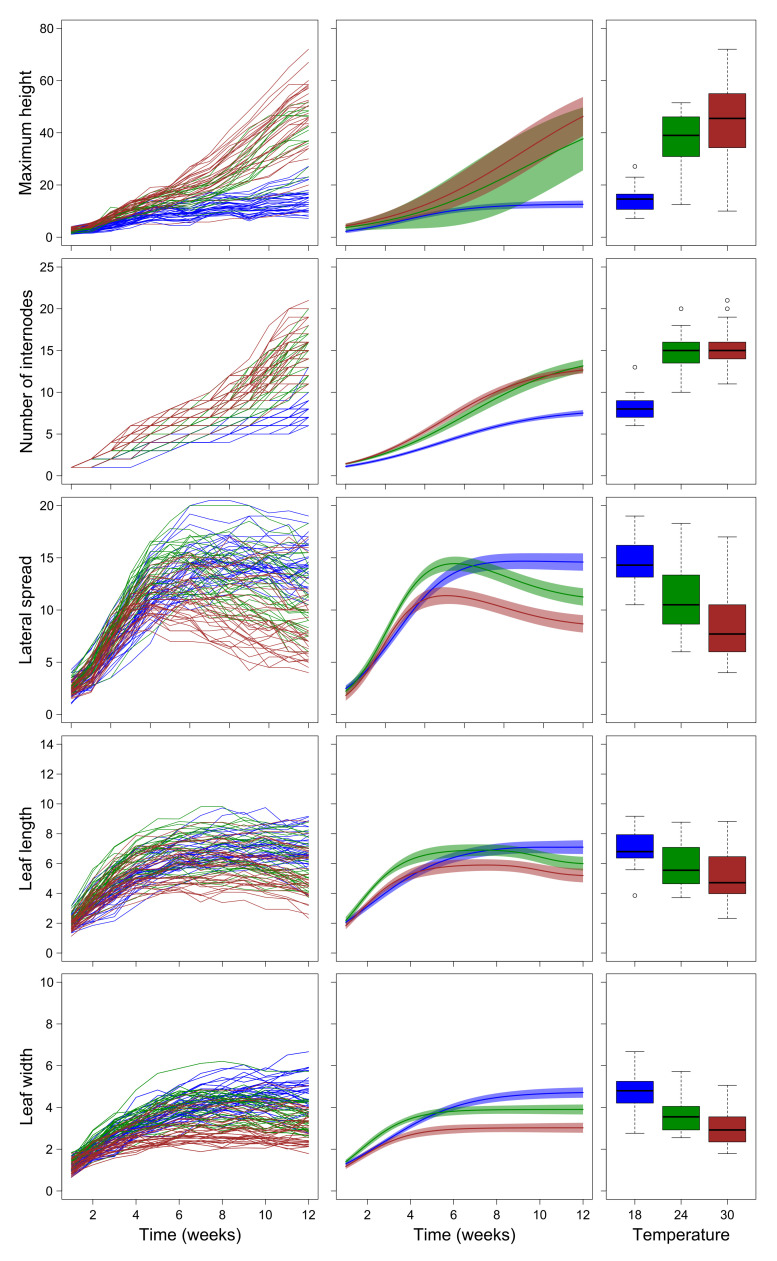
Left panels show growth trajectories of individual plants grown in the lab (after the removal of deviant data points). Each line represents an individual plant. Central panels show the interpolated growth curves and their 95% confidence intervals. Right panels show boxplots of the measured values of each plant at the last measure (week 12). Circles in the right panels represent outliers. In all panels, colors represent temperatures (blue = 18 °C, green = site 24 °C, red = 30 °C).

Maximum height and lateral spread of plants grown in the laboratory at different temperatures followed the same general growth pattern as plants grown in the field, as indicated by the fact that the same curves interpolated the data best (Table 3). Leaf length also showed a non-monotonic growth pattern, with a recession at later growth stages like plants grown in the field, but according to a slightly different parameterization of the double-Richards curves (compare Table 3 with Table 1). In contrast, in laboratory conditions, leaf width did not show the recession after the maximum value shown by plants grown in the field, although the growth trajectories of some individuals, particularly those grown at 18 °C, showed a decline at late times (see the left panel of Figure 3).

Generally, parameters describing growth trajectories of plants grown at 18 °C differed significantly from those of plants grown at 30 °C, while those of plants grown at 24 °C showed intermediate values. The only exceptions were parameters *i* and *s* of the growth trajectories of the number of internodes that did not differ significantly between 18 °C and 30 °C (Table 3). However, the general shape of the growth trajectory of plants grown at 24 °C was very similar to that of plants grown at 30 °C, while that of plants grown at 18 °C markedly differed (Figure 3). Generally, plants grown at 18 °C were shorter and with fewer internodes than those grown at 30 °C but had larger lateral spread and larger and longer leaves (Table 3, Figure 3). Plants grown at 24 °C showed a maximum height and a number of internodes similar to those of plants grown at 30 °C, while for lateral spread and leaf length and width they showed intermediate patterns between those of plants grown at 18 °C and 30 °C (Table 3, Figure 3).

### 2.3. Biomass and Reproductive Parameters of Plants Grown in Laboratory Conditions

**Figure 4:**
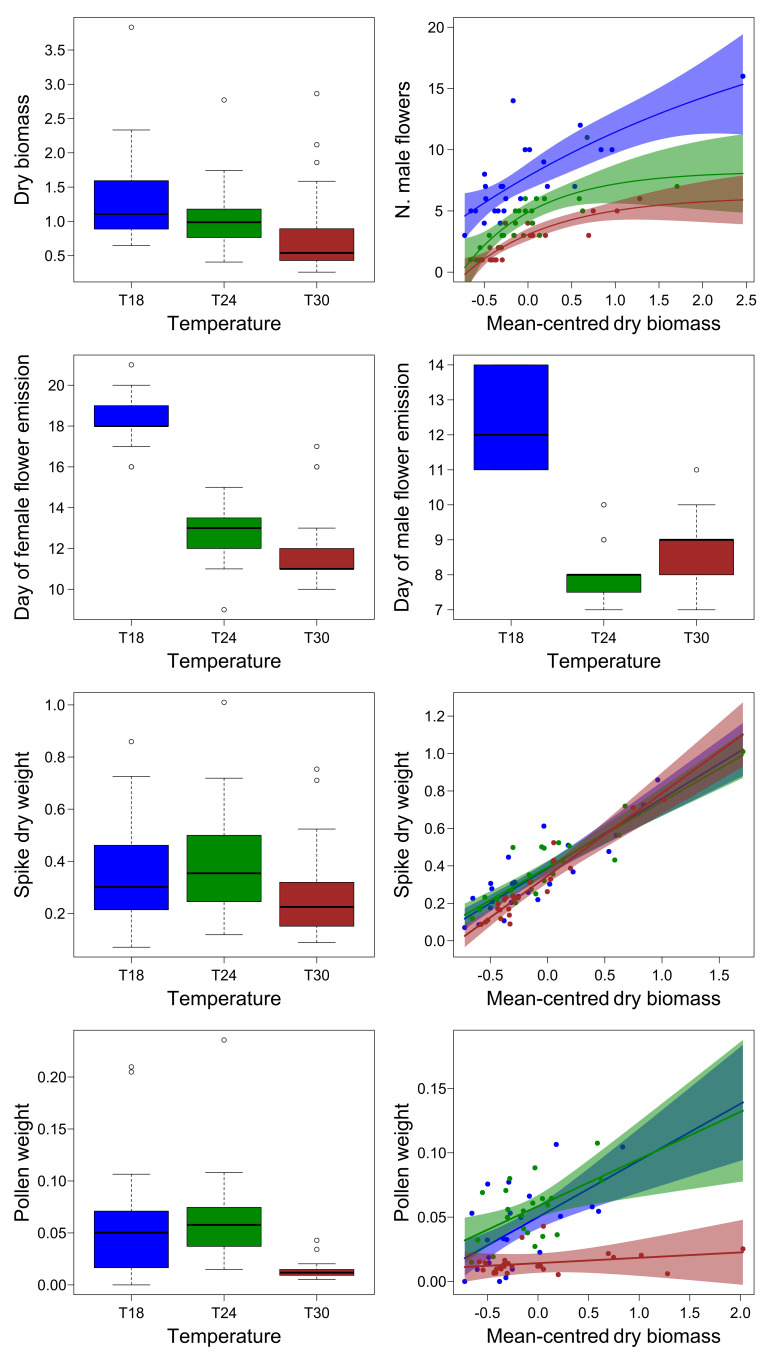
Boxplot and regression curves of dry biomass and reproductive parameters of plants grown in laboratory conditions. The shaded area represents the 95% confidence interval of the curve. Colors represent different temperatures (blue = 18 °C; green = 24 °C; red = 30 °C).

Dry biomass of plants grown in the laboratory was higher for plants grown at 18 °C than for plants grown at 30 °C, while plants grown at 24 °C showed intermediate values (Table 4, Figure 4).

Plants grown at 18 °C also emitted both male and female flowers later than those grown at 24 °C and 30 °C (Table 4, Figure 4). On the day of plant collection, both male and female flower emissions did not covary significantly with dry biomass (Table 4). Pollen weight was on average lower for plants grown at 30 °C than for those grown at lower temperature. In addition, pollen weight did not change with plant biomass for plants grown at 30 °C, while it increased significantly for those grown at 18 °C and 24° C. In contrast, spike dry weight increased significantly with plant dry biomass, but did not differ among growth temperatures (Table 4, Figure 4).

The number of male flowers changed non-linearly with plant biomass, following an asymptotic regression model (Table 5). Only parameter *L′* of this model differed significantly among temperatures. Since biomass values were centered within group before the analysis to reduce the collinearity among predictors (see Methods), the significant differences in L′ indicate that a plant of average size among those grown at 30 °C produced fewer male flowers that a plant of average size among those grown at 24 °C, and that this latter also produced significantly fewer male flowers than a plant of average size among those grown at 18 °C (Table 5, Figure 4).

## 3. Discussion

Our study revealed considerable phenotypic plasticity in terms of growth and reproductive performances of the invasive *Ambrosia artemisiifolia* L. (common ragweed) along altitude and temperature gradients, in field and controlled conditions. Overall, common ragweed reduced its size (plant height, stem, internodes, etc.) along the studied altitudinal gradient but with different strengths or patterns, especially when considering leaf traits. As a general rule, trait variability of common ragweed tended to reduce at higher altitude, likely due to environmental filters, as already observed for other alien species, and dissimilarly to what happens to native species that generally exhibit an increase of trait variability [28].

The ability of the common ragweed to modulate its phenotypic traits according to environmental gradients has been highlighted in previous studies performed along latitudinal (and temperature) gradients [39,40]. In addition, the modulation of traits in common ragweed within a single generation in response to increased temperatures in its invaded range has been recently explained as rapid evolution [41]. Previously, in the native range of the species, different “ecotypes” were observed to preadapt to local conditions, reducing plant height and increasing width in response to day length and temperature reductions [39].

Similarly, we found that plant height (stem, maximum height, and number of internodes) was the main contributor to the growth of common ragweed towards middle and low altitudes and the highest temperatures. Conversely, leaf traits increased in importance and size toward the highest altitude and the lowest temperatures.

With regard to the lab experiment, results showed similar growth trajectories to those observed in the field for plant height and leaf traits, but with contrasting trends. In fact, the species seems to mostly invest in vegetative vigor (i.e., biomass) and flower abundance at the lowest temperature tested (18 °C).

### 3.1. Field Experiment

In the field experiment, common ragweed exhibited different growth trajectories for traits related to height and leaf. Maximum height, stem height, and number of internodes grew according to a logistic curve over time, indicating that the plants elongate monotonically toward a plateau, with a trajectory likewise described for other species [42]. The plateau of the curve started in correspondence with the emission of flower buds (then removed in our study). Despite that we were not able to collect data on flowering time due to local authority restrictions, a previous study highlighted that the flowering phenology and growth pattern of all traits are associated with maximum plant height in herbaceous plant species [43].

The maximum value of plant height was not reached, as expected, at the lowest altitude of the gradient (site A: 130 m and 23.4 °C of mean temperature), but at the following growth station (i.e., site B: 250 m and 22.9 °C). With plant height being a key determinant of a species ability to compete for light [44], in the studied altitudinal gradient, this trait reached the maximum value at the site B, located at the base of the Prealpine slopes. Conversely, the minimum plant height was observed at the growth station with the highest altitude (site E: 1242 m and 16.6 °C of mean temperature) that also exhibited the lowest growth velocity.

On the other hand, leaf length, leaf width, and lateral spread (i.e., canopy) grew hump-shaped, according to a double-Richard curve, with a decrease of the trait value, likely in correspondence of the bud emission (then removed due to authority restrictions). In fact, previous authors observed that the maximum leaf size decreases early in the season, presumably prior to the reproductive onset [45,46]. Analogously, during the experiment, common ragweed tended to lose the lower leaves, indicating the tendency to allocate resource for reproduction tissues. In accordance with such observations, the timing of leaf senescence has been found to be in relation with the flowering phenology of numerous species along altitudinal gradients [47].

Leaf traits had better performances towards higher elevations (i.e., bigger size in relation to plant height) with lower mean temperatures, indicating that along the altitudinal gradient the species tends to invest more resources in photosynthesis and light capturing than in competition [48]. Indeed, larger individuals are expected to produce larger seeds, conferring higher competitive ability to seedlings [49,50]. This tendency is also mirrored in the leaf number. At the lowest and middle altitudes, common ragweed tended to maintain a higher number of smaller leaves.

All traits exhibited a tendency to reduce their variability along the altitudinal gradient. Similarly, the environmental constraints along altitudinal gradients (temperature, growth season, competitive ability, etc.) have been described to gradually limit the functional suitability of non-native species [29].

### 3.2. Laboratory Experiment

The laboratory experiment showed similar trends as the field one with respect to the growth trajectories (logistic and double-Richards curves) of common ragweed traits investigated at different temperature conditions. On the other hand, the reduction of the leaf traits (i.e., hump-shaped trajectory) along the observation period were less evident, probably due to the quite different light conditions of the growth chamber in comparison with those of the growth stations in the field. Additionally, in this case, a clear countertendency of the traits relating to height and those of leaves (lower temperatures) was observed along the temperature gradient: At the lowest temperatures (18 °C) the plants exhibited larger leaves and lower height, with the opposite trend at the highest temperatures (30 °C). In common ragweed, increasing temperature has been observed to increase transpiration rates [51]. An increased transpiration rate (and water loss) towards the highest temperatures can explain both the reduction of the leaf size and the lower biomass.

Concerning reproductive traits, our results indicated that at the lowest temperature common ragweed preserved its fitness (conservation of the pollen weight, highest number of male flowers, and same spike dry weight than the other temperatures), in turn supported by the highest biomass value. In this species, dry weight has already been observed to support reproductive performances at different growth conditions according to pH (i.e., inflorescence size and number of inflorescences; see [52]). These findings are surprising and in countertendency to those performed in the native range of the species, where common ragweed reduced its biomass and reproductive performance toward higher latitudes and lower temperatures [39]. In addition, taller individuals of common ragweed grown at the highest temperature (30 °C) tended to reach their maximum height and flowering (both male and female flowers) earlier than shorter ones; this trend is divergent with those observed by Sun and Frelich [43] on several herbaceous (native) grass species.

### 3.3. Implication for the Invasion Syndrome of Common Ragweed

The ability to reallocate biomass and plasticity in several phenotypic traits (e.g., plant height, number of leaves, etc.) is known to contribute to the invasion of alien species in new environments [53]. Our results are significant to understand how an invasive alien plant like the common ragweed may migrate and adapt to mountain ecosystems. Consequently, common ragweed would potentially compete with migrating native flora, since the distribution of mountain biota is moving upward in response to increasing temperature at the continental scales [54]. In any case, plants during migration are subject to new biotic and abiotic conditions that could favor selection in the migrating population of ragweed [41].

The difference in the trait growth trajectories and the shifts in biomass allocation found in the common ragweed at different altitudes and temperatures can certainly reflect its adaptation ability to new environmental conditions and, therefore, the potential to invade and compete for resources toward higher altitudes.

In the European invaded range of the species, the adaptability of its natural populations and their invasion potential are supported by high levels of intra-population genetic variation [34]. Whereas phenotypic plasticity may increase the ecological niche breadth of the species, post-introduction or post-colonization rapid evolution is known to produce genetically based phenotypic variations and adaptations, which can increase plant invasiveness. Therefore, phenotypic plasticity and rapid adaptation may be considered key components of the release–naturalization–invasion continuum [55]. In recent studies, common ragweed has been observed to grow in new areas at high altitude (over 1200 m) as a casual species; however, it seems that the species is not still able to establish at the current climatic conditions [38].

Although the plants seemed to have decreasing fitness (biomass) towards higher elevation, the laboratory experiment showed an opposite trend. In fact, the species conserved good fitness and reproductive ability in term of biomass and male and female flower production at low temperature. There are several explanations for these contrasting trends found in the field and laboratory experiments. First, findings of lab experiments need to be carefully assessed when they are translated to understand phenomena linked to natural populations [56]. Influences of environmental variables (abiotic and biotic) on the different responses between plant grown in the laboratory and in the field should be considered [57]. The increasing light intensity (especially UV-B radiation) at higher altitude may have limited plant size in terms of tissue formation and need for protecting the photosynthetic system with shorter individuals close to the soil [58]. Finally, the exposition of field plants to a variable regime of relative humidity and temperature peaks with drought periods may have played a role in shaping such differences. Indeed, during the last two decades, drought episodes have become stronger in terms of frequency and length [59] in the Po valley, with a subsequent increased evapotranspiration and lower relative humidity for the field plants. For this reason, in our study, the growth station at the lowest altitude could not be considered as optimal climatic conditions for developing biomass. Indeed, the better performances (in term of biomass and plant height) were found at the intermediate growth station (stations B and C) in the Prealps. This pattern may have repercussions in the future invasion trends of the species in hill/mountain areas currently presenting lower levels of disturbance (i.e., new colonization spaces) in comparison to the flat areas of the Po valley and, therefore, lower occurrence.

In perspective, our results can be used to inform future management decisions regarding common ragweed [60]. First, it is very likely that the expected increasing temperatures due to climate change will push plastic populations of the species to invade higher altitudes. So, to implement measures of early detection and eradication in the mountain areas falling within the invaded range of the species is recommended. On the other hand, considering possible biocontrol actions for the future, the onset of flowering at different temperature regimes will clearly set the limits of action of biocontrol agents of the species [26]. Indeed, if plants held at 24 °C and 30 °C opened their flowers at the same time, it would mean that for biocontrol agents like the ragweed leaf beetle *Ophraella communa*, there would be fewer day degrees available at 24 °C to build up high enough density population to suppress pollen release by common ragweed.

## 4. Material and Methods

### 4.1. Plant Material and Preliminary Germination

**Figure 5:**
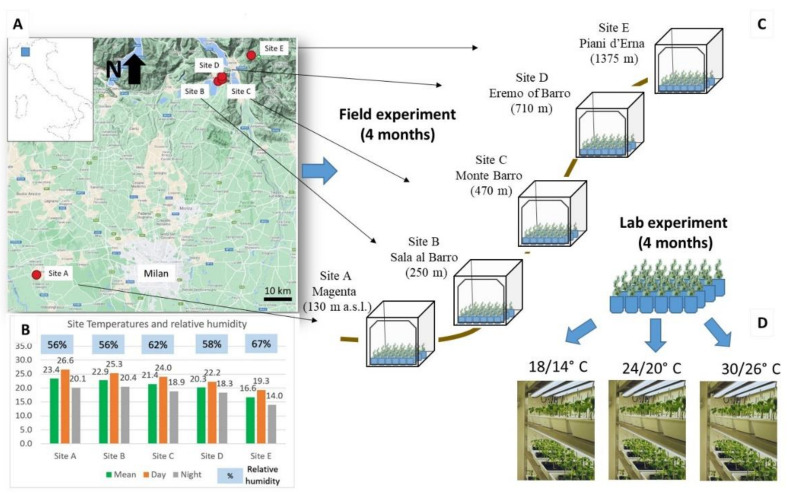
Experimental design of the study. (**A**) Study area of the field experiment and (**B**) mean temperatures and relative humidity of the selected sites (**C**) where a growth station of common ragweed (*A. artemisiifolia*) was set up. (**D**) In the laboratory experiment, common ragweed individuals were grown in three growth chambers set up at different temperature ranges (day/night): 18/14 °C, 24/20 °C, and 30/26 °C.

All the experiments were performed using common ragweed seeds collected from an agriculture area in Lombardy (45.597811 N; 8.869912 E). Seeds were cold-stratified at 4 °C for 3 months to overcome seed dormancy and then planted in a tray containing autoclaved natural soil for germination. Seedlings were transplanted in pots of 2.5 L and 0.75 L for the field and lab experiments, respectively (Figure 5). The pots contained the same standard soil made of potting soil (VigorPlant©, pH 6) and sand in the proportions of 60–40%.

### 4.2. Field Experiment

In order to study the trends of functional traits of common ragweed along an altitudinal gradient, five sites at increasing altitudes were selected in an area between the Po Valley and the Prealps in Lombardy, where the species has been established for several decades. As common ragweed is included in the regional blacklist of species to be controlled and whose spread is restricted [61], the following sites were selected in agreement with local authorities (Lombardy region, phytosanitary service): Site A at Magenta (130 m a.s.l.; lat. 45.459, lon. 8.874); site B at Sala al Barro (250 m; lat. 45.822, lon. 9.361); site C at the Monte Barro slope (470 m; lat. 45.825, lon. 9.372); site D at the Eremo of Barro (710 m; lat. 45.831, lon. 9.371); and site E at the Piani d’Erna (1242 m; lat. 45.870, lon. 9.449). Overall, the altitudinal gradient considered was about 1000 m.

At each site, a field cage with the species individuals was set up. Within the cages of 1 m^3^, 24 seedlings were grown in pots of 2.5 L for about 4 months, from June to September 2015. Each cage was placed in a flat area and covered with a soft insect net to avoid herbivory, especially by *Ophraella communa* (ragweed leaf beetle), which has been accidentally introduced in the region since 2013 and preferentially feeds on common ragweed [62]. The plants were watered weekly within plant pot saucers.

During plant growth, the following data about vegetative and reproductive traits were collected weekly: maximum plant height (cm), stem height (cm), number of internodes (*n*), lateral spread (cm; vertical projection of the area covered by the plant), number of leaves (*n*), leaf length (cm), and leaf width (cm). Measurements of the biomass of individuals over time were also conducted. To do this, six plants were removed from each site every 3 weeks and their dry and fresh weights of the aboveground portion were measured.

Due to the regional phytosanitary restrictions implemented to prevent allergic syndromes to human populations, flower buds were repeatedly removed from the plants during the flowering season in order to avoid the dispersion of the highly allergenic pollen of this species [32].

### 4.3. Lab Experiment

Three growth chambers with identical photoperiods, light intensity (15:9 h light:dark 150 μmol m^−2^ s^−1^) and humidity (65%) but different temperatures (LT: 18/14 °C light-dark, IT: 24–20 °C and HT: 30–26 °C) were set up. For each temperature, 51 seedlings within pots were grown for about 4 months (summer 2015), until seed production. During plant growth, the following data about vegetative and reproductive traits were collected weekly or at the end of the experiment: maximum plant height (cm), stem height (cm), number of internodes (*n*), lateral spread (cm), number of leaves (*n*), leaf length (cm) and leaf width, dry biomass at the end of the experiment (g), number of male flowers (*n*), day of male and female flower emission (*n* weeks of first flower open), spike dry weight (g), and pollen weight (g).

At maturity, pollen was collected from the plants by enveloping each spike with a transparent plastic collector, according to Ghiani et al. [32].

### 4.4. Data Analysis

Growth trajectories of morphological traits of common ragweed grown at different sites were modelled by fitting parametric growth curves with nonlinear mixed models (NLMMs) using the nlme procedure in the nlme package [63] of R.

Fitting parametric curves is computationally efficient and allows the estimation of parameters of biological significance [64]. In addition, NLMMs are very flexible statistical tools as they allow modelling any parameter of the growth curves as a function of different predictors. This flexibility extends also to the random part of the model because it is possible to enter different random structures for each parameter of the growth curve. However, fitting NLMMs is challenging. To reduce the complexity of these models, we ran preliminary analyses to assess (1) which growth curve best fitted the growth trajectory of the morphological trait under scrutiny and (2) which parameters of the growth curves showed large variability among individual plants, in order to properly parameterize the random part of the NLMMs.

Different morphological traits may show growth trajectories that can be described by different curves. Among the growth curves widely used in modelling plant growth trajectories [65,66,67], we selected five, including the double-Richards curves, which allow modelling growth trajectories for those morphological traits that change non-monotonically and decline after having reached a peak [64]. Equations for the five growth curves used in the analyses are reported in Table 6. To assess the growth curve that best fit the growth trajectory of each morphological trait, we fitted non-linear models (NLMs) to all data and compared the values of the small-sample Akaike’s information criterion (AICc) of each model. We note that these preliminary models did not account for repeated measures collected at each individual plant; however, we considered this approximation reasonable given the explorative nature of these analyses, which were only aimed at assessing the general shape of the growth trajectory of each morphological trait. The double-Richards curves were fitted with the *FlexParamCurve* v. 1.5-3 package [64].

To assess the structure of the random part of the NLMM, we followed the procedure described in Morganti et al. [68]. First, we interpolated the selected growth curves to data of each plant separately. Then, we plotted the range of parameters from curves fitted to individual plants and noted those that, at a visual inspection, showed large heterogeneity (see [68,69] for a similar approach). It should be noted that repeated measures of the same plant often showed temporal autocorrelation, and trait variance also usually increased with their size. In the final NLMMs, we, therefore, assumed (1) a random variation of those parameters showing large heterogeneity, (2) a first-order residual temporal autocorrelation, and (3) a change of the variance with time according to an exponential function, as suggested in Oswald et al. [64]. In the fixed part of the model, we allowed for variation of all model parameters among sites and, when we detected significant variation, we performed post-hoc tests for differences between each pair of sites with the Tukey method.

Growth curves of plants grown under laboratory conditions were fitted with the same procedure, but temperature (of the growth chambers) was included as a three-level predictor in the fixed part of the models, allowing for variation in all model parameters among temperatures.

Data collected in the field showed sometimes unreasonably large variations between consecutive measures that inflated non-linear model variance and caused convergence difficulties. We thus applied an in-home procedure (fully described in the supporting information) to remove deviant measures, which were replaced by missing values. Application of this procedure greatly improved non-linear model fit (details not shown).

Analyses of wet and dry biomass of plants were conducted using generalized least square (GLS) models that included as predictors: site (five-level factor), time (four-level factor), and the interaction among them for field-grown plants, while the predictors for laboratory-grown plants were temperature (three-level factor), time (four-level factor), and the interaction among them. The model also accounted for inhomogeneity of variances among groups, which was detected during preliminary model checks. Since each individual was measured only once in this part of the study, we entered no random term in the model.

From those laboratory-grown individuals that were kept until the end of the experiment, we also collected data on traits related to the dry biomass and the reproductive investment of the plant. These data were collected only at the endpoint of the experiment. Dry biomass was analyzed in an ANOVA model with temperature of the growth chamber (three-level factor) as predictor. Data on the reproductive investment of the plants were modelled using linear models (LMs) assuming a Gaussian error distribution with the temperature of the growth chamber (three-level factor), the dry biomass of the plant (covariate), and their interaction as predictors. Since dry biomass differed significantly among plants grown at different temperatures, we preliminarily centered dry biomass values while removing from the value of each plant the mean value of all the plants grown at that temperature. This procedure strongly reduced the collinearity among predictors (details not shown).

In the analysis of dry biomass, we removed potentially influential data (i.e., data that may strongly condition the results of the model) based on their Cook’s distance being >4/N, where N is the number of data [70]. In some cases, we also found small deviations from model assumptions. We, therefore, further checked significance of LMs by a randomization procedure performed with the *permuco* package [71], which, however, always confirmed the results of parametric tests (details not shown). The only exception was the number of male flowers that were modelled using an asymptotic growth curve in a NLM. Additionally, for these models, post hoc tests were performed with the Tukey method using the *emmeans* package [72].

All the analyses were performed in R 3.6.2 [73] with packages *FlexParamCurve* [64], *nlme* [67], *emmeans* [72], and *permuco* [71].

## 5. Conclusions

Common ragweed (*Ambrosia artemisiifolia* L.) was found to exhibit a high phenotypic plasticity in response to different altitude and temperature conditions. This ability may support future shifts of the species to higher altitude (and likely latitudes) under climate warming. In the face of climate change and the expected northward shift of the species [36], our study suggests a great ability of common ragweed to survive in a wide range of temperatures and the in situ resistance and adaptation of the natural populations currently abundant at low altitudes and latitudes in the European invaded range. Therefore, the expansion of the species toward higher altitude should be intensively monitored in the short and middle term. Our results are also directly relevant for projecting impact by *Ophraella communa* (ragweed leaf beetle) and probably other biocontrol agents.

In the near future, climate change may modify the altitudinal ranges of several invasive alien plants, increasing their impacts; therefore, control and management plans for plant invaders should take this aspect into account.

## Figures and Tables

**Table 1:** Final NLMMs of the growth trajectories of plants grown in field conditions. Coefficients of each parameter of the growth curves are reported in Table S1. The growth curve fitted and the number of observations and plants included in each analysis are reported as well as the residual degrees of freedom and the values of the temporal autocorrelation coefficient (φ) and of the Akaike information criterion corrected for a small sample size (AICc).

Parameter	F	df	*p*
Maximum height (three-parameter logistic curve, 867 observations and 89 plants)			
*K*	14.539	4	<0.001
*i*	1.7	4	0.148
*s*	1.914	4	0.162
Residual df = 764; φ = 0.578; AICc = 3702.3			
Stem height (three-parameter logistic curve, 502 observations and 65 plants)			
*K*	6.229	4	<0.001
*i*	2.281	4	0.060
*s*	4.192	4	0.002
Residual df = 423; φ = 0.609; AICc = 2242.9			
Number of internodes (four-parameter logistic curve, 849 observations and 89 plants)			
*L*	0.951	4	0.434
*K*	6.303	4	<0.001
*i*	0.483	4	0.748
*s*	0.432	4	0.785
Residual df = 741; φ = 0.558; AICc = 2438.2			
Lateral spread (double-Richards curve #31, 740 observations and 86 plants)			
*K*	11.949	4	<0.001
*r*	3.600	4	0.006
*i*	11.191	4	<0.001
*K′*	2.699	4	0.030
Residual df = 635; φ = 0.761; AICc = 2791.1			
*m* = 1.233, *r′* = 2.073, *i′* = 10, *m′* = 0.963			
Number of leaves (double-Richards curve #31, 453 observations and 59 plants)			
*K*	1.379	4	0.241
*r*	1.864	4	0.116
*i*	1.035	4	0.389
*K′*	4.844	4	0.001
Residual df = 375; φ = 0.737; AICc = 3050.7			
*m* = −0.123, *r′* = 1.438, *i′* = 12, *m′* = 0.944			
Leaf length (double-Richards curve #34, 773 observations and 89 plants)			
*K*	6.145	4	<0.001
*r*	1.677	4	0.086
*i*	0.104	4	0.606
*r′*	2.271	4	0.229
*i′*	1.283	4	0.275
Residual df = 660; φ = 0.637; AICc = 1351.5			
*i* = −0.924, *K′* = −4.645, *m′* = 0.880			
Leaf width (double-Richards curve #31, 773 observations and 89 plants)			
*K*	12.171	4	<0.001
*r*	2.047	4	0.086
*i*	0.68	4	0.606
*K′*	1.14	4	0.229
Residual df = 665; φ = 0.792; AICc = 855.3			
*m* = 0.938, *r′* = 2.580, *i′* = 10, *m′* = 0.819			

**Table 2:** GLS models of the wet and dry biomass according to time, site, and their interaction.

Parameter	F	df	*p*
Wet biomass (*n* = 120)			
Time	38.318	3	<0.001
Site	2.464	4	0.05
Time × Site	3.087	12	0.001
Dry biomass (*n* = 120)			
Time	51.068	3	<0.001
Site	2.755	4	0.032
Time × Site	4.143	12	<0.001

**Table 3:** Final NLMMs of the growth trajectories of plants grown in laboratory conditions. Coefficients of each parameter of the growth curves are reported in Table S2. The growth curve applied and the number of observations and plants included in each analysis are reported as well as the residual degrees of freedom and the values of the autocorrelation coefficient (φ) and of the Akaike information criterion corrected for small sample size (AICc).

Parameter	F	df	*p*
Maximum height (three-parameter logistic curve, 982 observations and 76 plants)			
*K*	83.642	2	<0.001
*i*	78.978	2	<0.001
*s*	25.544	2	<0.001
Residual df = 898; φ = 0.904; AICc = 3974.1			
Number of internodes (three-parameter logistic curve, 1096 observations and 85 plants)			
*K*	64.147	2	<0.001
*i*	5.007	2	0.007
*s*	3.484	2	0.003
Residual df = 741; φ = 0.558; AICc = 2438.2			
Lateral spread (double-Richards curve #31, 1182 observations and 96 plants)			
*K*	16.213	2	<0.001
*r*	26.439	2	<0.001
*i*	17.121	2	<0.001
*K′*	13.381	2	<0.001
Residual df = 1075; φ = 0.726; AICc = 3398.5			
*m* = 1.228, *r′* = 0.542, Ri = 7.739, *m′* = 1.000			
Leaf length (double-Richards curve #31, 453 observations and 59 plants)			
*K*	11.727	2	<0.001
*r*	49.162	2	<0.001
*i*	16	2	<0.001
*K′*	7.138	2	0.001
Residual df = 997; φ = 0.704; AICc = 1376.1			
*m* = 0.572, *r′* = 1.372, *i′* = 10, *m′* = 0.998			
Leaf width (three-parameter logistic curve, 1119 observations and 96 plants)			
*K*	46.151	2	<0.001
*i*	48.659	2	<0.001
*s*	27.652	2	<0.001
Residual df = 1015; φ = 0.615; AICc = 421.4			

**Table 4:** Linear models of biomass and reproductive parameters of plants grown in laboratory conditions except for the number of male flowers. Coefficients of each parameter of the growth curves are reported in Table S3. Number of plants included in each analysis is reported as well as the residual degrees of freedom and the value of the Akaike information criterion corrected for small sample size (AICc).

Parameter	F	df	*p*
Dry biomass (76 plants)			
Temperature	4.841	2	0.011
Residual df = 73; AICc = 148.4			
Day of emission of female flowers (71 plants)			
Temperature	105.800	2	<0.001
Centred dry biomass	0.421	2	0.519
Temp. x c. dry biomass	0.007	2	0.992
Residual df = 65; AICc = 277.8			
Day of emission of male flowers (72 plants)			
Temperature	110.462	2	<0.001
Centred dry biomass	2.851	2	0.096
Temp. x c. dry biomass	1.027	2	0.364
Residual df = 66; AICc = −225.7			
Spike dry weight (72 plants)			
Temperature	1.872	2	0.162
Centered dry biomass	294.893	2	<0.001
Temp. x c. dry biomass	1.347	2	0.267
Residual df = 65; AICc = −141.6			
Pollen weight (70 plants)			
Temperature	34.639	2	<0.001
Centered dry biomass	24.125	2	<0.001
Temp. x c. dry biomass	7.147	2	<0.001
Residual df = 64; AICc = −345.0			

**Table 5:** Final NLM of the number of male flowers produced by plants grown in laboratory under different temperature conditions. The growth curve applied and the number of plants included in the analysis are reported as well as the residual degrees of freedom and the value of the Akaike information criterion corrected for small sample size (AICc). Coefficients of each parameter of the growth curves are reported in Table S4.

Parameter	F	df	*p*
Number of male flowers (Asymptotic regression 73 plants)			
*K*	0.75	2	0.477
*L′*	37.75	2	<0.001
*r*	1.211	2	0.305
Residual df = 64; AICc = 237.3.8			

**Table 6:** Equations and parameters of the five growth curves used in the analyses. The morphological traits whose growth trajectory was best modelled by each curve are also reported. All curves are function of time (*t*) except for the asymptotic curve, which was used to model the number or male flowers of plants grown under laboratory conditions and was a function of plant dry biomass (*x*). See Figure S1 for an illustration of these parameters.

Growth Curve	Equation	Morphological Trait
(1) Linear	$y=y_{0}+rt$	Reproductive parameters except for the number of male flowers
(2) Asymptotic	$y=K+\left(L^{\prime }-K\right)e^{-rx}$	Number of male flowers
(3) Three-parameter logistic	$y=\frac{K}{1+e^{\frac{i-t}{s}}}$	Plant height, stem height
(4) Four-parameter logistic	$y=L+\frac{K-L}{1+e^{\frac{i-t}{s}}}$	Number of leaves
(5) Double-Richards	$y=\frac{K}{{\left(1+me^{r\left(i-t\right)}\right)}^{\frac{1}{m}}}+\frac{K^{\prime }}{{\left(1+m^{\prime }e^{r^{\prime }\left(i^{\prime }-t\right)}\right)}^{\frac{1}{m^{\prime }}}}$	Lateral spread, leaf length, leaf width
Parameter	Description	
$y_{0}$	Intercept, corresponding to mean value if t is centred	
*r* and *r′*	Growth rates	
*s*	Scale parameter replacing the growth rate r (s=1/r) in the parameterization of Equations (2) and (3) of *SSlogis* and *SSfpl* (used to fit them)	
*K*	Upper asymptote	
*L and L′*	Lower asymptote or initial value	
*m* and *m′*	Shape parameters of the generalized logistic curves, values > 1, imply that the inflection points are realized sooner than *i* or *i′* and the growth rates at *i* or *i′* are lower than *r* or *r′*; values < 1 imply the opposite	
*i* and *i′*	Inflection points, i.e., time at which the fastest growth/recession is attained	
*K′*	Difference between asymptotes of the curve before and after recession	

## Supplementary Materials

The following are available online at https://www.mdpi.com/article/10.3390/plants10102144/s1, Table S1: Final NLMMs of the growth trajectories of plants grown in field conditions, Table S2: Final NLMMs of the growth trajectories of plants grown in laboratory conditions, Table S3: Linear models of biomass and reproductive parameters of plants grown in laboratory conditions except for the number of male flowers, Table S4: Final NLM of the number of male flowers produced by plants grown in laboratory under different temperature conditions.
