# Supplementary material for: High Phenotypic Plasticity in a Prominent Plant Invader along Altitudinal and Temperature Gradients
Source: Plants (Basel). 2021 Oct 9;10(10):2144. doi: 10.3390/plants10102144 (PMC8538053; doi:10.3390/plants10102144)
Supplement: Supplementary file 1 [file plants-10-02144-s001.zip › plants-1400688-supplementary.pdf]

plants

Supporting information for

High Phenotypic Plasticity in a Prominent Plant Invader along Altitudinal and Temperature Gradients

Rodolfo Gentili [1,*], Roberto Ambrosini [2], Benno Augustinus [3], Sarah Caronni [1], Elisa Cardarelli [1], Chiara Montagnani [1], Heinz Müller-Schärer [4], Urs Schaffner [5], Sandra Citterio [1]

[1] Department of Earth and Environmental Sciences, University of Milano-Bicocca, Piazza della Scienza 1, I-20126 Milano, Italy; rodolfo.gentili@unimib.it, sarah.caronni@unimib.it, elisa.cardarelli@unimib.it, chiara.montagnani@unimib.it, sandra.citterio@unimib.it
[2] Department of Environmental Science and Policy, University of Milan, Via Celoria 26, I-20133 Milano, Italy; roberto.ambrosini@unimi.it
[3] Swiss Federal Institute for Forest, Snow and Landscape Research WSL, Zuercherstrasse 111, CH-8903 Birmensdorf, Switzerland; benno.augustinus@wsl.ch
[4] Department of Biology, University of Fribourg, Chemin du Musée 10, CH-1700 Fribourg, Switzerland; heinz.mueller@unifr.ch
[5] CABI, Rue des Grillons 1 CH-2800 Delémont, Switzerland; u.schaffner@cabi.org
* Correspondence: rodolfo.gentili@unimib.it; Tel.: +39 02 64482700

Citation: Gentili, R.; Ambrosini, R.; Augustinus, B.; Caronni, S.; Cardarelli, E.; Montagnani, C.; Müller-Schärer, H.; Schaffner, H.; Citterio, S; High Phenotypic Plasticity in a Prominent Plant Invader along Altitudinal and Temperature Gradients. *Plants* **2021**, *10*, 2144. https://doi.org/10.3390/plants10102144

Academic Editor: Firstname Lastname

Received: date
Accepted: date
Published: date

Publisher's Note: MDPI stays neutral with regard to jurisdictional claims in published maps and institutional affiliations.

Copyright: © 2021 by the authors. Submitted for possible open access publication under the terms and conditions of the Creative Commons Attribution (CC BY) license (https://creativecommons.org/licenses/by/4.0/).

Table S1: Final NLMMs of the growth trajectories of plants grown in field conditions. Coefficients of each parameter of the growth curves are shown separately for all parameters that differed significantly among sites. Parameters are reported in Table 6. Different low-case letters in the contrast column denote coefficients that differed significantly at post-hoc tests. The growth curve fitted and the number of observations and plants included in each analysis is reported as well as the residual degrees of freedom, the value of the autocorrelation coefficient (φ) and thet of the Akaike information criterion corrected for small sample size (AICc). For double-Richards curves, we also report the values of the parameters that did not show variation and therefore were fixed to the mean value among all individuals (see Oswald et al. **[41]**).

Parameter	F	df	P	Site	Coef.	S.E.	Contr.
Maximum height (three-parameter logistic curve, 867 observations and 89 plants)							
K	14.539	4	< 0.001	A	60.889	2.766	a
				B	61.427	2.281	a
				C	64.215	2.736	a
				D	48.361	3.243	b
				E	36.626	0.234	b
i	1.700	4	0.148	A	8.166	0.372	
				B	7.246	0.339	
				C	8.475	0.365	
				D	8.157	0.468	
				E	7.992	0.596	
s	1.914	4	0.162	A	2.498	0.135	
				B	2.227	0.127	
				C	2.619	0.132	
				D	2.646	0.161	
				E	2.758	0.206	
Residual df = 764, φ = 0.578, AICc = 3702.3							

Table S1: Continued.

Parameter	F	df	P	Site	Coef.	S.E.	Contr.
Stem height (three-parameter logistic curve, 502 observations and 65 plants)							
K	6.229	4	< 0.001	A	43.008	2.633	a
				B	47.402	3.376	a
				C	46.942	2.263	a
				D	40.528	4.535	ab
				E	30.595	2.680	b
i	2.281	4	0.060	A	7.117	0.253	
				B	6.665	0.290	
				C	7.303	0.211	
				D	8.200	0.518	
				E	7.693	0.346	
s	4.192	4	0.002	A	1.778	0.093	cd
				B	1.606	0.110	ce
				C	1.991	0.069	d
				D	2.163	0.182	cd
				E	2.202	0.140	d
Residual df = 423, φ = 0.609, AICc = 2242.9							
Number of internodes (four-parameter logistic curve, 849 observations and 89 plants)							
L	0.951	4	0.434	A	-4.988	2.331	
				B	-4.154	1.611	
				C	-5.125	2.073	
				D	-3.842	1.801	
				E	-1.769	1.020	
K	6.303	4	< 0.001	A	21.825	2.988	ab
				B	23.143	2.145	a
				C	22.6231	2.754	a
				D	19.848	2.238	ab
				E	13.6898	1.079	b
i	0.483	4	0.748	A	7.117	0.253	
				B	6.665	0.290	
				C	7.303	0.211	
				D	8.200	0.518	
				E	7.693	0.346	
s	0.432	4	0.785	A	1.778	0.093	
				B	1.606	0.110	
				C	1.991	0.069	
				D	2.163	0.182	
				E	2.202	0.140	
Residual df = 741, φ = 0.558, AICc = 2438.2							

Table S1: Continued.

Parameter	F	df	P	Site	Coef.	S.E.	Contr.
Lateral spread (double Richards curve #31, 740 observations and 86 plants)							
K	11.949	4	< 0.001A	15.579	0.523	ab	
				B	18.215	0.536	c
				C	16.504	0.482	ac
				D	13.752	0.480	b
				E	14.242	0.513	b
r	3.600	4	0.006	A	1.473	0.102	d
				B	1.398	0.089	d
				C	1.391	0.092	d
				D	2.119	0.181	e
				E	1.568	0.123	de
i	11.191	4	< 0.001	A	2.594	0.079	f
				B	2.675	0.075	f
				C	2.702	0.076	f
				D	2.560	0.068	f
				E	3.192	0.077	g
K'	2.699	4	0.030	A	-0.684	1.017	h
				B	-3.939	1.055	hi
				C	-4.092	0.860	hi
				D	-4.767	0.858	ji
				E	-4.371	0.951	hi
Residual df = 635, φ = 0.761, AICc = 2791.1							
m = 1.233, r' = 2.073, i' = 10, m' = 0.963							

Table S1: Continued.

Parameter	F	df	P	Site	Coef.	S.E.	Contr.
Number of leaves (double Richards curve #31, 453 observations and 59 plants)							
K	1.379	4	0.241	A	59.018	4.945	
				B	77.567	7.715	
				C	57.510	7.349	
				D	61.038	4.807	
				E	56.368	5.244	
r	1.864	4	0.116	A	0.570	0.085	
				B	0.355	0.078	
				C	0.374	0.092	
				D	0.618	0.083	
				E	0.491	0.077	
i	1.035	4	0.389	A	3.925	0.134	
				B	3.518	0.243	
				C	3.857	0.310	
				D	4.020	0.118	
				E	4.098	0.176	
K'	4.844	4	0.001	A	-9.030	5.433	a
				B	-33.127	6.012	b
				C	-18.621	6.735	ab
				D	-39.528	5.130	b
				E	-31.511	5.094	b
Residual df = 375, φ = 0.737, AICc = 3050.7							
m = -0.123, r' = 1.438, i' = 12, m' = 0.944							

Table S1: Continued.

Parameter	F	df	P	Site	Coef.	S.E.	Contr.
Leaf length (double Richards curve #34, 773 observations and 89 plants)							
K	6.145	4	< 0.001	A	9.499	0.375	a
				B	11.649	0.432	b
				C	10.616	0.641	ab
				D	8.592	0.549	a
				E	9.257	0.601	a
r	1.677	4	0.086	A	0.404	0.033	
				B	0.343	0.025	
				C	0.318	0.034	
				D	0.394	0.046	
				E	0.297	0.034	
i	0.104	4	0.606	A	1.483	0.058	
				B	1.503	0.054	
				C	1.515	0.063	
				D	1.535	0.062	
				E	1.522	0.073	
r′	2.271	4	0.229	A	0.949	0.140	
				B	0.864	0.121	
				C	0.632	0.105	
				D	0.551	0.095	
				E	0.575	0.089	
i′	1.283	4	0.275	A	10.442	0.270	
				B	10.271	0.305	
				C	10.829	0.665	
				D	11.751	0.763	
				E	9.682	0.576	
Residual df = 660, φ = 0.637, AICc = 1351.5							
i = -0.924, *K′* = -4.645, *m′* = 0.880							

Table S1: Continued.

Parameter	F	df	P	Site	Coef.	S.E.	Contr.
Leaf width (double Richards curve #31, 773 observations and 89 plants)							
K	12.171	4	< 0.001	A	4.752	0.158	a
				B	5.470	0.187	b
				C	4.716	0.156	a
				D	4.254	0.150	ac
				E	3.906	0.148	c
r	2.047	4	0.086	A	0.960	0.064	
				B	0.928	0.059	
				C	0.794	0.055	
				D	0.752	0.060	
				E	0.855	0.068	
i	0.680	4	0.606	A	3.091	0.104	
				B	3.236	0.104	
				C	3.270	0.127	
				D	3.106	0.146	
				E	3.278	0.138	
K'	1.140	4	0.229	A	-1.551	0.265	
				B	-1.304	0.345	
				C	0.873	0.230	
				D	-1.124	0.209	
				E	-0.826	0.218	
Residual df = 665, φ = 0.792, AICc = 855.3							
m = 0.938, r' = 2.580, i' = 10, m' = 0.819							

Table S2: Final NLMMs of the growth trajectories of plants grown in laboratory conditions. Coefficients of each parameter of the growth curves are shown separately for all parameters that differed significantly among sites. Parameters are reported according to Table 6. Different letters denote coefficients that differed significantly at post-hoc tests. The growth curve applied and the number of observations and plants included in each analysis is reported as well as the residual degrees of freedom, the value of the autocorrelation coefficient (φ) and that of the Akaike information criterion corrected for small sample size (AICc). For double-Richards curves, we also report the values of the parameters that did not show variation and therefore were fixed to the mean value among all individuals (see Oswald et al. **[41]**).

Parameter	F	df	P	Site	Coef.	S.E.	Contr.
Maximum height (three-parameter logistic curve, 982 observations and 76 plants)							
K	83.642	2	< 0.001	T = 18	12.686	0.750	a
				T = 24	54.173	7.6695	b
				T = 30	64.549	4.288	b
i	78.978	2	< 0.001	T = 18	2.841	0.250	c
				T = 24	9.118	0.967	d
				T = 30	8.863	0.453	d
s	25.544	2	< 0.001	T = 18	1.846	0.158	e
				T = 24	3.493	0.311	f
				T = 30	3.373	0.169	f
Residual df = 898, φ = 0.904, AICc = 3974.1							
Number of internodes (three-parameter logistic curve, 1096 observations and 85 plants)							
K	64.147	2	< 0.001	T = 18	8.040	0.280	a
				T = 24	14.741	0.766	b
				T = 30	13.288	0.502	b
i	5.007	2	0.007	T = 18	4.885	0.261	c
				T = 24	6.151	0.350	d
				T = 30	4.942	0.222	c
s	3.484	2	0.003	T = 18	2.683	0.150	ef
				T = 24	2.762	0.145	e
				T = 30	2.343	0.101	f
Residual df = 741, φ = 0.558, AICc = 2438.2							

Table S2: Continued

Parameter	F	df	P	Site	Coef.	S.E.	Contr.
Lateral spread (double Richards curve #31, 1182 observations and 96 plants)							
K	16.213	2	< 0.001	T = 18	15.390	0.632	a
				T = 24	16.274	0.512	a
				T = 30	12.513	0.458	b
r	26.439	2	< 0.001	T = 18	0.734	0.032	c
				T = 24	0.985	0.036	d
				T = 30	1.110	0.046	d
i	17.121	2	< 0.001	T = 18	2.616	0.117	e
				T = 24	2.131	0.072	f
				T = 30	1.826	0.071	g
K'	13.381	2	< 0.001	T = 18	-0.857	0.704	h
				T = 24	-5.520	0.575	i
				T = 30	-4.221	0.498	i
Residual df = 1075, φ = 0.726, AICc = 3398.5							
m = 1.228, *r'* = 0.542, *i'* = 7.739, *m'* = 1.000							
Leaf length (double Richards curve #31, 453 observations and 59 plants)							
K	11.727	2	< 0.001	T = 18	7.298	0.225	a
				T = 24	6.962	0.200	a
				T = 30	5.961	0.191	b
r	49.162	2	< 0.001	T = 18	0.522	0.026	c
				T = 24	0.904	0.035	c
				T = 30	0.866	0.037	d
i	16.000	2	< 0.001	T = 18	2.180	0.099	e
				T = 24	1.555	0.053	f
				T = 30	1.603	0.060	f
K'	7.138	2	0.001	T = 18	-0.166	0.179	g
				T = 24	-1.031	0.148	h
				T = 30	-0.813	0.133	h
Residual df = 997, φ = 0.704, AICc = 1376.1							
m = 0.572, *r'* = 1.372, *i'* = 10, *m'* = 0.998							

Table S2: Continued

Parameter	F	df	P	Site	Coef.	S.E.	Contr.
Leaf width (three-parameter logistic curve, 1119 observations and 96 plants)							
K	46.151	2	< 0.001	T = 18	4.747	0.131	a
				T = 24	3.903	0.127	b
				T = 30	3.027	0.121	c
i	48.659	2	< 0.001	T = 18	2.812	0.105	d
				T = 24	1.663	0.083	e
				T = 30	1.549	0.093	e
s	27.652	2	< 0.001	T = 18	1.856	0.081	f
				T = 24	1.138	0.061	g
				T = 30	1.187	0.074	g
Residual df = 1015, $\varphi = 0.615$, AICc = 421.4							

Table S3: Linear models of biomass and reproductive parameters of plants grown in laboratory conditions except for the number of male flowers. Least square mean values and coefficients are shown separately for each temperature. Different low-case letters in the contrast column denote coefficients that differed significantly at post-hoc tests. Number of plants included in each analysis is reported.

Parameter	F	df	P	Temperature	Coef.	S.E.	Contr.
Dry biomass (76 plants)							
Temperature	4.841	2	0.011	T = 18	1.371	0.122	a
				T = 24	1.065	0.126	ab
				T = 30	0.838	0.122	b
Residual df = 73, AICc = 148.4							
Day of emission of female flowers (71 plants)							
Temperature	105.800	2	< 0.001	T = 18	18.348	0.333	a
				T = 24	12.729	0.328	b
				T = 30	11.963	0.334	b
Centred dry biomass	0.421	2	0.519				
Temp x c. dry biomass	0.007	2	0.99	T = 18	-0.316	0.751	
				T = 24	-0.221	0.659	
				T = 30	-0.204	0.554	
Residual df = 65, AICc = 277.8							
Day of emission of male flowers (72 plants)							
Temperature	110.462	2	< 0.001	T = 18	12.371	0.227	a
				T = 24	7.957	0.230	b
				T = 30	8.615	0.213	b
Centred dry biomass	2.851	2	0.096				
Temp x c. dry biomass	1.027	2	0.364	T = 18	-0.600	0.332	
				T = 24	-0.751	0.642	
				T = 30	0.004	0.337	
Residual df = 66, AICc = -225.7							
Spike dry weight (72 plants)							
Temperature	1.872	2	0.162	T = 18	0.350	0.017	
				T = 24	0.357	0.017	
				T = 30	0.302	0.018	
Centred dry biomass	294.893	2	< 0.001				
Temp x c. dry biomass	1.347	2	0.267	T = 18	0.372	0.037	
				T = 24	0.351	0.034	
				T = 30	0.442	0.045	
Residual df = 65, AICc = -141.6							

Table S3: Continued

Parameter	F	df	P	Temperature	Coef.	S.E.	Contr.
Pollen weight (70 plants)							
Temperature	34.639	2	< 0.001	T = 18	0.045	0.004	a
				T = 24	0.055	0.004	a
				T = 30	0.014	0.004	b
Centred dry biomass	24.125	2	< 0.001				
Temp x c. dry biomass	7.147	2	< 0.001	T = 18	0.044	0.010	e
				T = 24	0.037	0.013	ef
				T = 30	0.004	0.006	f
Residual df = 64	AICc = -345.0						

Table S4: Final NLM of the number of male flowers produced by plants grown in laboratory under different temperature conditions. Coefficients of each parameter of the growth curves are shown separately. Parameters are reported according to Table 6. Different low-case letters in the contrast column denote coefficients that differed significantly at post-hoc tests. The growth curve applied and the number of plants included in the analysis is reported as well as the residual degrees of freedom and the value of the Akaike information criterion corrected for small sample size (AICc).

Parameter	F	df	P	Temperature	Coef.	S.E.	Contr.
Number of male flowers (Asymptotic regression 73 plants)							
K	0.750	2	0.477	T = 18	24.239	21.599	
				T = 24	8.246	2.036	
				T = 30	6.174	1.495	
L′	37.750	2	< 0.001	T = 18	7.824	0.551	a
				T = 24	4.878	0.400	b
				T = 30	3.066	0.253	c
r	1.211	2	0.305	T = 18	0.248	0.370	
				T = 24	1.171	0.684	
				T = 30	0.987	0.534	
Residual df = 64, AICc = 237.3.8							

1. Oswald, S.A.; Nisbet, I.C.T.; Chiaradia, A.; Arnold, J.M. FlexParamCurve: R package for flexible fitting of nonlinear parametric curves. *Methods Ecol. Evol.* **2012**, *3*, 1073-1077.
